# Perceived abusive supervision and graduate students’ suicidal ideation: from the perspective of interpersonal psychological theory of suicide

**DOI:** 10.1186/s40359-023-01136-z

**Published:** 2023-03-27

**Authors:** Yingying Yao, Fangbai Dong, Zhihong Qiao

**Affiliations:** 1 Counselling and Education Centre, Student Affairs Department, Xiamen University, Xiamen, Fujian China; 2 Faculty of Psychology, Beijing Normal University, Beijing, China; 3 Student Mental Health Center, Henan Agricultural University, Zhengzhou, Henan China

**Keywords:** Abusive supervision, Suicidal ideation, Thwarted belongingness, Perceived burdensomeness, Supervisor–student relationship

## Abstract

**Background:**

In recent years, the issue of suicide caused by the stress of a contradictory relationship between graduate students and academic supervisors has aroused heated discussion in society. Based on the interpersonal psychological theory of suicide, this study aims to examine the influence of the perceived abusive supervision on graduate students’ suicidal ideation and the parallel mediating roles of thwarted belongingness and perceived burdensomeness.

**Methods:**

We conducted a cross-sectional online survey measuring perceived abusive supervision, interpersonal psychological needs and suicidal ideation among 232 Chinese graduate students. A structural equation model was constructed to test the hypothesis.

**Results:**

The results showed that abusive supervision directly exacerbated suicidal ideation (β = 0.160, 95% CI = [0.038, 0.281], *p* = 0.009) and indirectly influenced suicidal ideation through thwarted belongingness (β = 0.059, 95% CI = [0.008, 0.110], *p* = 0.019) and perceived burdensomeness (β = 0.102, 95% CI = [0.013, 0.191], *p* = 0.018). The indirect effect accounted for 50.15% of the overall effect.

**Conclusions:**

These findings enrich our understanding of the influence of supervisor–student relationship by integrating the literatures on educational and organizational behaviour, and provide practical insights for psychosocial interventions from the perspective of the interpersonal psychological theory of suicide.

## Background

Over the past decade, a growing number of researchers have turned their attention to the severe problems associated with suicide among college students [1]. In particular, the suicide vulnerability of graduate students has come under the spotlight recently [2]. A study conducted in the United States confirmed the elevated suicide risk of graduate students, finding that 21.2% of graduate students met the criteria for suicide risk and 15% of them reported that they had "seriously considered attempting suicide" [3]. Similarly, among Chinese graduate students, 25.7% of them had ever thought about or attempted to kill themselves and 6.3% had the suicidal ideation in past one year [2]. This is a huge group that needs urgent attention but has received limited attention to before.

Research has found that interpersonal relationships have a huge impact on one’s mental health [4]. For graduate students, the relationship with the supervisor is the most critical, if not the most central, interpersonal relationship. In recent years, the suicide problem caused by the stress of a poor relationship between graduate students and their academic supervisors has aroused a heated discussion in society [5]. As an adverse form of supervisor–student relationships, whether and how does abusive supervision affect graduate students' suicidal ideation is an important starting point of this study.

### Abusive supervision

Abusive supervision refers to persistent hostile verbal and non-verbal behaviours perceived by subordinates from their supervisors, excluding physical contact [6]. Typical behaviours include teasing and verbal abuse, such as blaming, put-downs, and name-calling [7]. As dysfunctional leadership behaviours, abusive supervision has been widely studied due to its ubiquity in the workplace and its negative impact on subordinates and organizations. A literature review showed that abusive supervision was negatively associated with subordinates’ attitudes, behaviours, relationships and overall well-being [8]. For example, abusive supervision had a negative effect on organizational citizenship behaviour [9], organizational commitment [10] and performance [11], increased emotional exhaustion [12] and turnover intention [13]. Abusive supervision is a widespread but intractable social issue that may exist beyond formal employment relationships [14].

We noticed that a common phenomenon in universities that graduate students assist their supervisors in completing research projects, and most graduate students can receive a regular stipend or salary from their supervisors in return. Supervisors have the discretion to provide resources and support for their graduate students’ research progress, and even to decide whether a student can ultimately graduate [15]. The relationship between the supervisor and the graduate student has gradually changed from an equal relationship to another one, which is similar to the relationship between the boss and the subordinate in the workplace [16]. As the Chinese saying goes "Once a teacher, always a father", what the teacher says and does are considered to be the moral code and guidance for graduate students. Culture is the lens through which individuals observe and interpret social interactions [12]. Chinese Confucian Cultural environment makes the "paternalistic or authoritarian leadership" to be one of the most common management styles of academic supervisors. So, we conducted studies in a higher education setting to examine the effect of perceived abusive supervision on suicidal ideation among graduate students. Abusive supervision in the higher education setting refers to the students’ perception of the extent to which the academic supervisors consistently displays verbal and non-verbal hostile behaviours, excluding physical contact [15].

### Abusive supervision and suicidal ideation

Abusive supervision is an interpersonal stressor that causes tension and leads to negative short-term (proximal) and long-term (distal) outcomes [17, 18]. The resource conservation theory (COR theory) posits that stress occurs (a) when key resources are threatened with loss, (b) when essential resources are lost, or (c) when there is a failure to gain resources following significant effort [17, 18]. Based on COR theory, individuals possess and accumulate valuable psychological resources which they can use to adapt, resist or overcome threats. When confronted by abusive supervision, an individual has to invest more effort, leading to a high stress [19]. The persistent lack of resources may lead to severe psychological loss and consequent stress, ultimately negatively impacting mental health [20] and increasing suicidal risk [21]. As suggested by the transactional model of stress and coping [22], stressors are considered risks to mental health when external demands exceed the resources an individual has. Long-term exposure to high pressure will cause individuals to enter the exhaustion stage, and lead to emotional symptoms such as depression and frustration, and in extreme cases self-destruction and suicidal tendencies [23].

The effect of negative life events on mental health has been verified in a large number of practical studies [24, 25]. Abusive supervision is one of the most destructive stressors with adverse consequences for subordinates and has been shown to be significantly positively associated with suicidal ideation [26, 27], and even one of the predictors of suicidal ideation in the workplace [26–28]. Abusive supervision is strongly negatively correlated with suicidal ideation through some proximal variables, such as depression from the perspective of the affective event theory [28], and unmet basic psychological needs from the perspective of the Self-determination theory [27] and loss of meaning in life [26]. Despite this, the studies on the direct association between abusive supervision and the suicidal ideation remains fairly limited.

Studies on college students have shown that abusive supervision increases students' anxiety about their research projects and diminishes their mental health and program satisfaction [4]. Abusive supervision further severely affects their intrinsic motivation [16], reduces academic creativity [16] and leads to maladaptive behaviour such as academic procrastination [29, 30]. If a supervisor abuses a graduate student, the chronic lack of guidance and scientific support and the inevitable interpersonal stress that followst. We proposed that a potential detrimental outcome of abusive supervision is a risk of suicidal ideation among graduate students. Based on theory and previous findings, we hypothesized a conceptual link between abusive supervision and suicidal ideation in graduate students (Hypothesis1).

### Abusive supervision and thwarted belongingness

As an empirically proven theory of suicide risk, the Interpersonal Psychological Theory of Suicide (IPTS) proposes that the human need to belong is a basic need that, if not met, may have negative mental health consequences, such as an increased rates of suicidal ideation throughout the life cycle [31]. IPTS suggests two related interpersonal needs, thwarted belongingness and perceived burdensomeness [32].

Thwarted belongingness is characterized by feelings of loneliness and the absence of reciprocal care, which includes a variable mental state of psychological distress that may be influenced by external interpersonal interactions [33]. As a kind of social destructive behaviour and hostility expression, abuse supervision affects social interpersonal communication. In the workplace, the interpersonal interactions of authority figures affect one's psychological connection with peers or groups, that is the leaders’ behaviours can elevate (or weaken) an employee’s belongingness by supporting (or undermining) his status in the group [34]. Some studies based on the COR theory have found out that abusive supervision may lead to interpersonal withdrawal [35] and interaction avoidance [12].

Abusive supervision as a social disruptive behaviour influence the likelihood and degree of depression in employees and accelerate the germination of depression through persistent emotional influence derived from affective interpersonal experiences [28]. Studies carried out in different settings have shown that the abusive supervision weakens an individual's attachment to the organization [11] and is negatively correlated with the satisfaction of belonging needs [34]. It is also verified that the satisfaction of belonging needs plays a mediating role in the negative relationship between abusive supervision and safety behaviour [34]. Subordinates actively reduce direct contact with their superiors [36] or even actively distance themselves from the supervisors [6] in the abusive environment. The need for belonging may be difficult to satisfy when encountering abusive supervision. Therefore, we hypothesized that abusive supervision was associated with graduate students’ thwarted belongingness (Hypothesis 2a).

### Abusive supervision and perceived burdensomeness

Perceived burdensomeness refers to a feeling of inefficiency or incompetence, which is composed of two dimensions: a perception of self-hatred and the belief that suicide can liberate others. According to social cognitive theory, environmental factors are either obstacles to or promoters of efficacy beliefs. Abusive supervision, a dysfunctional relationship, could be environmental barriers to a person's self-efficacy, which refers to one’s confidence to accomplish tasks and the motivational force to facilitate his/ her behaviours [37]. Abusive supervisors find it difficult to recognize subordinates’ achievements [38] and tend to ignore subordinates’ achievements [39]. According to social cognitive theory, positive feedback is an important source of self-efficacy. Studies have revealed that abusive supervision made subordinates feel undervalued [40], reduced one’s self-efficacy at work [41] and challenged subordinates’ occupational adaptation ability by destroying professional self-efficacy [42].

According to the broad definition and classification of resources in COR theory, a good working environment and comfortable interpersonal relationships can be divided into personal resources and energic resources. When students face abusive supervision, it means not only a lack of subjective resources, but also a lack of objective guidance which is essential to graduate academic development. The more abusive supervision one experiences, the less resources he or she will receive from the supervisor, making academic research more difficult and further disappointing the supervisor. Correspondingly, the self-efficacy of graduate students decreases while the sense of burdensomeness increases. We hypothesized that abusive supervision is positively correlated with perceived burdensomeness in graduate students (Hypothesis 2b).

### Abusive supervision, interpersonal psychological needs and suicidal ideation

The two interpersonal psychological needs proposed in IPTS, thwarted belongingness and perceived burdensomeness, are considered to be proximal variables of suicidal ideation, while stressful life events and other risk factors are relatively distal in the causal chain for suicide [33]. High levels of thwarted belongingness and perceived burdensomeness are associated with suicidal ideation [32] which is supported by empirical research [43]. A qualitative comparative analysis assessing the occurrence of suicide attempts found that the high-risk suicide group reported a higher level of thwarted belongingness and perceived burdensomeness [43]. A meta-analysis [31] of studies testing IPTS found that the deprivation of thwarted belongingness and perceived burdensomeness were positively associated with suicidal ideation [31, 44] each of these factors independently predicted suicidal ideation [45].

Abusive supervision makes it more difficult to maintain interpersonal relationships [20], which may further damage the ability to meet one’s interpersonal needs, with a range of adverse consequences, including increased risk of suicide [31]. Although no direct studies have been conducted on the relationship between abusive supervision and interpersonal psychological needs, some studies have focused on the consequences of abusive supervision, prompting us to verify the possible mediating roles of thwarted belongingness (Hypothesis3a) and perceived burdensomeness (Hypothesis 3b).

### Present study

This study focused on the relationship between perceived abusive supervision and suicidal ideation. On the basis of IPTS, the mediating roles of interpersonal psychological needs were proposed in the model of theoretical hypothesis. In this study, we proposed a hypothetical mediation model with three hypotheses. First, abusive supervision would be positively associated with suicidal ideation. Second, abusive supervision would significantly influence the two components of interpersonal psychological needs (thwarted belongingness and perceived burdensomeness). Finally, both thwarted belongingness and perceived burdensomeness would mediate the relationship between abusive supervision and suicidal ideation.

## Methods

### Participants and sampling

We conducted a cross-sectional study using an online survey of graduate students from two comprehensive universities. Convenient sampling and snowball sampling were used to collect data through questionnaire links. The recruitment requirements for the participants are as follows: (1) able to read Chinese; (2) spent at least one semester with his/her supervisor. 243 patients participated in this study, and 11 participants were excluded because they did not complete the questionnaire, with a completion rate of 95.47%. The final sample included 232 participants, 61.6% of whom were women. They ranged in age from 22 to 33 (Mage = 23.45, SD = 1.99).

Participants were informed about the purpose and procedure of the study prior to the survey. Online written consents were obtained from all participants. This study was approved by the Research Ethics Review Committee of Beijing Normal University.

### Measures

#### Perceived abusive supervision

This study used a 10-item Chinese version of perceived Abuse Supervision scale [46], adapted from Tepper’s (2000) original Abusive Supervision scale [7]. The adapted version retains the culturally neutral items of the original scale and is more appropriate for a college and university context. The scale is a subjective assessment of graduate students' perceptions of sustained hostile verbal or non-verbal supervisory behaviours in the supervisor–student relationship. For example, “my supervisor ridicules me” and “my supervisor tells me my thoughts or feelings are stupid”. The ten items were rated on 5-point scale, from 1 (never) to 5 (always). The internal consistency in the study was 0.859. We computed Comparative Fit Index (CFI), Tucker–Lewis Index (TLI), Root Mean Square Error of Approximation (RMSEA), and Standardized Root Mean Residual (SRMR) as indicators of structure validity. And the structure validity was acceptable (CFI = 0.930, TLI = 0.895, *RMSEA* = *0.106,* SRMR = 0.052).

#### Interpersonal psychological needs

Thwarted belongingness and perceived burdensomeness were assessed by the Interpersonal Needs Questionnaire using a 15-items self-report scale [33] which was validated in Chinese in 2018 [47]. The 9-item thwarted belongingness subscale includes items such as “These days, I feel like I belong” and “These days, I often feel like an outsider in social gatherings”. The perceived burdensomeness subscale includes 7 items, including “These days I think I make things worse for the people in my life”. Items were scored on a 7-point Likert scale ranging from 1 (not at all true for me) to 7 (very true for me), with higher scores indicating a deficiency in meeting interpersonal psychological needs. In the current study, the internal consistency of the two subscales were 0.868 and 0.941, respectively. The one-level and two-dimension structural validity was good (CFI = 0.930, TLI = 0.905, *RMSEA* = *0.100,* SRMR = 0.076) in the sample.

#### Suicidal ideation

Suicidal ideation was assessed using a brief version of Beck Scale for Suicide Ideation [48]. A total of 5 questions were used to assess suicidal ideation, with a score from 0–2 for each item. Sample items include “wish to live”, “wish to die” and “reasons for living or dying”. The total score for suicidal ideation is the sum of these five items. Higher scores reflect a higher likelihood of suicidal ideation and risk. The internal consistency in the study was 0.925, and the structural validity was good in the sample (CFI = 0.980, TLI = 0.960, *RMSEA* = *0.100,* SRMR = 0.025). In addition, a self-designed question was used to directly discriminate whether the participants had suicidal ideation: "Have you ever had suicidal thoughts in the past year", with two choices (0 = no, 1 = yes).

#### Demographic variables

Participants were also asked to report the age, gender and Socioeconomic Status (SES) as the covariates in our study. SES was measured using Engel coefficient, which is a proportion of total food spending. the proportion of food expenditure in total expenditure. There were five response choices ranging from 1 = poor (above 59%), 2 = be with adequate food (50–59%), 3 = relatively well-off (40–50%), 4 = well-off (30–40%), 5 = richest (below 30%).

### Data analysis

Descriptive statistics reported demographic characteristics and variables of interest. Correlational analyses were used to test bivariate associations between variables. The continuous variables (age, abusive supervision, thwarted belongingness, perceived burdensomeness and suicidal ideation) were not normally distributed. Spearman correlation analysis was used. A Mann–Whitney *U* test was used for age comparison, and a Chi-square test was used for gender and SES comparison. The hypothesized mediation model was examined by structural equation modeling (SEM). To evaluate the fit degree of the model, we computed the normed χ^2^, CFI, TLI, RMSEA and SRMR as indicators of model fitness [49]. The goodness-of-fit indices are as follows: both CFI and TFI parameters are 0.90 or above, RMSEA and SRMR are 0.08 or less. In addition, we tested the significance of the mediating effect using a 95% confidence interval (CI) bootstrap method from 5000 samples. Two-tailed significance level was set at 0.05. SPSS 23.0 and Mplus 8.0 were used for the above data analysis.

## Results

### Common method bias test

Harman univariate analysis was performed for common methodological biases on all items of the Abuse Supervision Scale, Interpersonal Needs Questionnaire, and Beck Scale for Suicide Ideation. Results showed no significant methodological bias [50] for there were 15 factors with eigenvalues greater than 1, with the first factor accounted for 26.10% of the variance and less than the critical value of 40%.

### Background characteristics and covariates

The final sample consisted of 232 participants and Table 1 showed the background characteristics of them. No significant differences were found between participants with and without suicidal ideation on demographic characteristics (i.e., gender, age, or SES). More than a third of the participants reported suicidal ideation in the past year.

**Table 1:** Demographic characteristics of the study sample (N = 232)

		With suicidal ideation (n = 85) n (%)	Without suicidal ideation (n = 147) n (%)	*p* value
Gender	Male (n = 89)	26 (30.6%)	63 (42.9%)	0.287
	Female (n = 143)	59 (69.4%)	84 (57.1%)	
Mean Age (SD)		24.56 ± 1.70	24.56 ± 2.15	0.281
SES	Poor (n = 15)	4 (4.7%)	11 (7.5%)	0.398
	Be with adequate food (n = 96)	34 (40.0%)	62 (42.5%)	
	Relatively well-off (n = 104)	43 (50.6%)	61 (41.8%)	
	Well-off (n = 12)	4 (4.7%)	8 (5.5%)	
	Richest (n = 4)	0 (0%)	4(2.7%)	

Descriptive statistics of the variables and the correlation matrix of their relationships are presented in Table 2. The mean frequency of abusive supervision among the graduate students is 1.75 ± 0.75, which is similar to other studies conducted among graduate students in the China [16, 51]. Spearman's correlation test was used to analyse the correlations among abusive supervision, thwarted belongingness, perceived burdensomeness and suicidal ideation. Suicidal ideation was significantly associated with abusive supervision (*r* = 0.38, 95% CI = [0.27, 0.49], *p* < 0.001), thwarted belongingness (*r* = 0.44, 95% CI = [0.32, 0.55], *p* < 0.001) and perceived burdensomeness (*r* = 0.45, 95% CI = [0.33, 0.57], *p* < 0.001). Abusive supervision was significantly associated with thwarted belongingness (*r* = 0.35, 95% CI = [0.22, 0.46], *p* < 0.001) and perceived burdensomeness (*r* = 0.26, 95% CI = [0.13, 0.38], *p* < 0.001).

**Table 2:** Correlation between main variables (N = 232)

	M ± SD	1	2	3	4	5	6	7
1 Gender	–	1.00						
2 Age	24.56 ± 1.99	0.09	1.00					
3 SES	2.52 ± 0.82	0.08	− 0.15*	1.00				
4 Abusive supervision	1.75 ± 0.75	0.07	0.17*	0.00	*0.859*			
5 Thwarted belongingness	2.47 ± 1.02	0.06	− 0.09	− 0.14*	.35***	*0.868*		
6 Perceived burdensomeness	1.62 ± 0.96	0.03	− 0.24***	− 0.07	.26***	0.53***	*0.941*	
7 Suicidal ideation	3.00 ± 2.54	− 0.02	− 0.07	− 0.06	.38***	0.44***	0.45***	*0.925*

The sex was coded as “0 = male, 1 = female”, *p < 0.05, ***p < 0.001. Italics indicate the scales internal consistency coefficients

### Mediating effects of interpersonal psychological needs

We used path analysis to test model, which included abusive supervision as a predictor, suicidal ideation as a dependent variable, and three demographic variables thought to influence suicidal ideation, gender, age [52] and socioeconomic status [53], controlled as covariates. After controlling for covariates, abusive supervision was found to have a significant main effect on suicidal ideation (*β* = 0.332, 95% CI = [0.175, 0.490], *p* < 0.001). The results indicated that the more abusive supervision a person experienced, the more likely he or she was to have suicidal ideation.

We performed SEM to compare the two mediating mechanisms before interpreting the path coefficients. Both alternative models used abusive supervision as a predictor of suicidal ideation and were assessed using gender, age and socioeconomic status as covariates. In Model 1, the latent variable interpersonal psychological needs is the only mediator, and thwarted belongingness and perceived burdensomeness are indicators, for they are the subscales of INQ and there is significant correlation between them. However, Model 2 takes thwarted belongingness and perceived burdensomeness as two parallel mediators, and the fitting indexes of the two models are shown in Table 3.

**Table 3:** Model fit statistics for alternative models

	*χ*^2^*/df*	*CFI*	*TLI*	*RMSEA*	*SRMR*
Model1: IPN as latent variable mediator	17.673	0.931	0.827	0.125	0.044
Model2: TB and PB as parallel mediators	15.43	0.962	0.905	0.077	0.044

*IPN* interpersonal psychological needs; *TB* thwarted belongingness; *PB* perceived burdensomeness

Table 3 shows Model 1 has a poor model fitting (χ2*/df* = 17.673, *CFI* = 0.931, *TLI* = 0.827, *RMSEA* = 0.125, *SRMR* = 0.044) when compared with Model 2, indicating that the two paths from thwarted belongingness and perceived burdensomeness to suicidal ideation were unequal. Model 2 with thwarted belongingness and perceived burdensomeness as parallel mediators fit the data better (*χ*^2^*/df* = 15.43, CFI = 0.962, TLI = 0.905, RMSEA = 0.077, SRMR = 0.044).

**Fig. 1:**
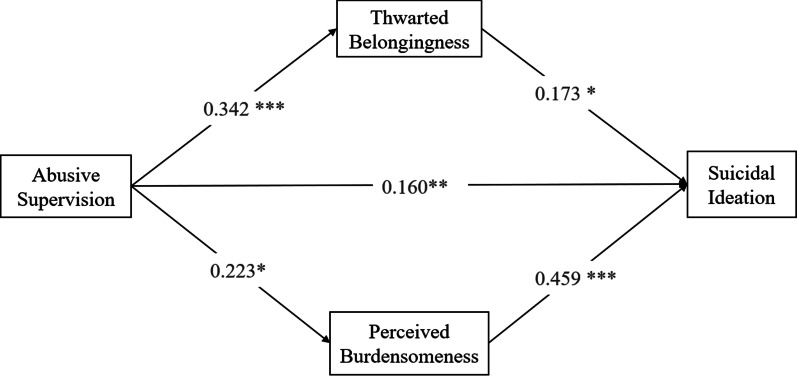
Model of indirect effect of abusive supervision on suicidal ideation through thwarted belongingness and perceived burdensomeness

Path analysis results of the hypothetical model showed that after controlling for covariates, abusive supervision was positively associated with thwarted belongingness (*β* = 0.342, 95% CI = [0.210, 0.474], *p* = 0.010) and perceived burdensomeness (*β* = 0.223, 95% CI = [0.051, 0.394], *p* = 0.011). Thus, hypothesis 2a and hypothesis 2b, in which abusive supervisors positively predicted graduate students’ thwarted belongingness and perceived burdensomeness, were supported. Additionally, thwarted belongingness (*β* = 0.173, 95% CI = [0.037, 0.309], *p* = 0.013) and perceived burdensomeness (*β* = 0.459, 95% CI = [0.328, 0.588], *p* < 0.001) significantly positively predicted suicidal ideation (Fig. 1).

The mediating roles of thwarted belongingness and perceived burdensomeness were also found in the hypothetical model. The Bootstrapping results of the mediating effects are displayed in the Table 4. The results demonstrated that abusive supervision predicted a high level of suicidal ideation through one direct path (*β* = 0.160, 95% CI = [0.038, 0.281], *p* = 0.009), as well as two indirect paths, one through thwarted belongingness (*β* = 0.059, 95% CI = [0.008, 0.110], *p* = 0.019), and the other through perceived burdensomeness (*β* = 0.102, 95% CI = [0.013, 0.191], *p* = 0.018). There was a significant correlation between abusive supervision and suicidal ideation among graduate students. Interpersonal psychological needs (thwarted belongingness and perceived burdensomeness) partially mediated this process. The indirect effect accounted for 50.15% of the overall effect. Therefore, hypothesis 3a and hypothesis 3b were also supported.

**Table 4:** Bootstrapping results for mediating effects

Effects	Pathways	Effect size	*p* value	95% confidence interval	
				Lower limit	Upper limit
Direct effect	AS–SI	0.160	0.009	0.038	0.281
Mediation paths	AS–TB–SI	0.059	0.019	0.008	0.110
	AS–PB–SI	0.102	0.018	0.013	0.191
Total indirect effect		0.161	0.001	0.058	0.264
Total effect		0.321	0.000	0.166	0.476

*AS* abusive supervision; *TB* thwarted belongingness; *PB* perceived burdensomeness; *SI* suicidal ideation

## Discussion

First of all, our study did find the phenomenon of abusive supervision in the relationship of academic supervisors and graduate students. The frequency of abusive supervision was similar to other studies conducted among the graduate students [16, 51], but far less than abusive supervision took place the workplace [42, 54, 55]. In Chinese culture, the tradition of respecting teachers and upholding morality was put forward as early as the Southern Dynasty, and what teachers said and did was regarded as the moral line and guidance for graduate students. Graduate students with power distance orientation are more likely to respond to supervisors through silence, for high-power distance orientation has been proven to moderate the relationships between them [17, 55] and may increase the perceived acceptability of abusive supervision. We suspect that there may be some differences between eastern and western cultures, mainly due to differences in power distance and the orientation of mentor roles. As far as we know, our study is the first one to focus on the impact of abusive supervision on mental health issue under the setting of higher education and we call for more attention to abusive supervision in teacher–student relationships.

There were two other main findings from our research. First, our study found a positive correlation between abusive supervision and suicidal ideation among graduate students. Our findings were consistent with previous studies suggesting that abusive supervision predicted detrimental outcomes for subordinates [17], exacerbated emotional exhaustion [12] and was associated with depression [28]. In particular, a poor relationship between a supervisor and the graduate student has been identified as a main stressor, and research have shown a consistent negative association between the quality of such relationship and suicidal ideation [26, 28, 36]. The relationship can be explained through the COR theory and social exchange theory. Material exchange (e.g., reward) and social interaction (e.g., encouragement, support, prestige) were reduced when students perceived abusive supervision, including indifference, sarcasm and even criticism and demeaning comments. Students further exhibit negative exchange behaviours (such as avoiding direct contact), resulting to poor quality of the supervisor–student exchange relationships. Some studies indicated that abusive supervision was positively related to turnover intentions of subordinates [40, 56]. However, abusive supervision, where it exists, can be a persistent phenomenon in the supervisor–student relationship, as it is very difficult to change supervisors in China. The arrested flight model suggests that a person will commit suicide because they can neither face nor escape social problems [57].When faced with desperate situations, some aggressive and irrational behaviours will be displayed [20].

Secondly, from the perspective of interpersonal psychological theory of suicide, we found that thwarted belongingness and perceived burdensomeness were the parallel mediators of the relationship between perceived abusive supervision and suicidal ideation in graduate students. COR theory holds that the lack of appropriate resources makes individuals more vulnerable to further loss of resources, leading them to suffer from a vicious cycle of depletion [20]. Abusive supervision reduces an individual's attachment to the supervisor and commitment to the group [11, 56], leading to an exacerbation of thwarted belongingness. From the perspective of social exchange theory, the more abusive supervision one experiences, the less likely exchanging behaviour is to occur [58]. Specifically, in the academic field, the support from supervisors is an important resource for graduate students. At the same time, the negative attitude and evaluation from the supervisor often lead to students' negative evaluation of their own abilities and interpersonal relationships, resulting in frustration in learning, lowered self-efficacy [42], and increased sense of burdensomeness. Our results also support the hypothesis of the interpersonal psychological theory of suicide that thwarted belongingness and perceived burdensomeness are key pathways to suicidal ideation.

However, when interpersonal psychological needs were deemed as a latent variable, the model fit was poor, and differences were found in the effects of thwarted belongingness and perceived burdensomeness. A large part of the supervisor–student interaction is the discussion on students' academic performance. As the main evaluator of students' achievements, the supervisors’ attitude can easily be interpreted as an evaluation of their abilities. Long-term abusive supervision will reduce students' self-efficacy and induce a sense of burdensomeness. This is consistent with IPTS-based studies, that is, perceived burdensomeness has a more significant impact on suicide tendencies [59, 60].

Our research has several theoretical implications. First, we examined the correlation between abusive supervision and suicidal ideation by integrating the literature on educational and organizational behaviour. This has enriched our understanding of the relationship between perceived abusive supervision and suicidal ideation among Chinese graduate students, a topic that has not been studied before. Second, we identified interpersonal psychological needs as a potential mechanism for understanding how abusive supervision leads to suicidal ideation. These results provide empirical support for the interpersonal psychological theory of suicide. The results suggested that preventive measures that enhance the sense of belonging and reduce perceived burdensomeness may inhibit the further development of suicidal ideation.

Research also has important implications for educational practice. Firstly, it serves as a warning that failing to recognize and deal with the adverse effects of abusive supervision can seriously damage the supervisor–student relationship [26] and the mental health of graduate students. Ministry of Education of China issued the " Code of conduct for the Supervisors of Graduate Students" in 2020, which proposed that first responsibility of supervisors is to cultivate postgraduates. In line with our findings, supervisors are also have a duty to care about the mental health of graduate students and to establish a good interaction mechanism. Supervisors should consciously strive to eliminate and control abusive supervision, in order to avoid its negative effects. Supervisors are encouraged to provide timely and practical support to students facing stress or failure rather than mocking them or belittling them. Secondly, other coping measures should be explored to alleviate the adverse effects of abuse supervision on interpersonal psychological needs. For example, emotional regulation efforts can be an effective way for students to maintain acceptable relationships with their supervisors [19].The relationships of supervisors and graduate students are complex. A strict management methods of supervisors such as the establishment higher standards to facilitate learning can benefit students' scientific research and innovation performance at moderate intensity [46]. To guide students to view the management of supervisors objectively and pluralistically may be benefit to reduce the negative impact of perceived abusive supervision. Besides, social supports from family or research team can also cushion the suicide risk of graduate students. Thirdly, in Chinese culture, the belief that excellent students are trained by strict teachers may lead to stricter guidance to promote students' achievements. As the academic authority of students, the evaluation of supervisors is an important source of students' academic self-efficacy. Supervisors are encouraged to identify students' strengths in a more approachable way. When students fail to complete the assigned research tasks satisfactorily, helping students to identified problems and summarize experiences would improve supervisor–student relationships and student learning efficiency, as well as reduce the risk of suicide among graduate students.

There are also some suggestions for colleges and universities. A good communication platform is conducive to improving the supervisor–student relationship and promoting students’ mental health. Therefore, colleges and universities are encouraged to improve the feedback channels for students' opinions and suggestions. In addition, universities should provide resources and support such as counselling and well-being workshops for graduate students to cushion the negative effect of abusive supervision and other forms of maladaptive supervising behaviours. Finally, universities should also focus on improving the emotional intelligence and interpersonal skills of supervisors and graduate students.

## Limitations and future directions

Despite these highlights, our findings suggest that much work remains to be done. First, a cross-sectional study precludes us from making causal inferences about variables. Therefore, longitudinal study and experimental design should be considered for future studies. Second, while self-reporting is one of the most common methods of assessing abusive supervision, the actual extent of abusive behaviours by supervisors is still unclear [19]. In future research, it would be more helpful and appropriate to add objective indicators to measure abusive supervision in the supervisor–student relationship. Third, perceived abusive supervision was used as the antecedent variable in our study. Future studies could extend the model one step further by examining what triggers abusive supervision. Do factors in the organization still play a role in the academic field? For example, it would be important to better understand the impact of personal traits factors, such as attachment orientation or social efficacy [61], environmental factors, such as stress or organizational climate [62, 63] and interaction factors [64, 65] between supervisor and graduate students. Supervisors should not only give academic guidance to students, but also care about their professional and personal development. Universities should strengthen the restraint and management of supervisors. From the perspective of graduate students, understanding what factors will affect their perception of abusive supervision is also an important topic. The literature on trauma provides substantial support for the finding that the effects of abusive supervision can be greatly influenced by an individual’s perceptual processes and history [17]. Individual differences (such as attribution styles) play an essential role in the evaluation process, and universities should provide guidance to students about how to better receive advice or suggestions for academic improvement from supervisors more objectively. Finally, cultural factors could influence the relationship between abusive supervision and mental health outcomes and deserve to be explored in future research. For example, the difference in interpersonal psychological needs between collectivism culture and individualism cultures, would affect the role of thwarted belongingness and perceived burdensomeness in the relationship chain. In addition, power dynamics play a central role in understanding supervisor–graduate relationships, as power distance orientation influences how graduate students perceive, evaluate, and react to abusive supervisory behaviours [12]. Meanwhile, abusive supervision decreased help-seeking behaviour and the willingness to provide direct or indirect help [66], reducing an individual's sense of connection. Therefore, we call for more research to discover the influence of cultural factors and to examine the differential effects of individual-level factors (e.g., power-distance orientation) and population-level factors (e.g., individualism-collectivism).

## Conclusions

This study found evidence for the effect of perceived abusive supervision on suicidal ideation among graduate students and the interpersonal psychological needs, thwarted belongingness and perceived burdensomeness, as parallel mediators aggravate suicidal ideation. That is abusive supervision was one of the risk factors for suicidal ideation among graduate students by imposing a barrier to meet interpersonal psychological needs.

## Data Availability

Data supporting the results of this study are available from Y.Y., the corresponding author. The data cannot be made public because it contains information that could compromise the privacy of study participants.
